# Supplementary material for: Exercise Reduces Glucose Intolerance, Cardiac Inflammation and Adipose Tissue Dysfunction in Psammomys obesus Exposed to Short Photoperiod and High Energy Diet
Source: Int J Mol Sci. 2024 Jul 15;25(14):7756. doi: 10.3390/ijms25147756 (PMC11277119; doi:10.3390/ijms25147756)
Supplement: Supplementary file 1 [file ijms-25-07756-s001.zip › ijms-3063802-supplementary.pdf]

Supplemental Figure S1

(a) **(b)**

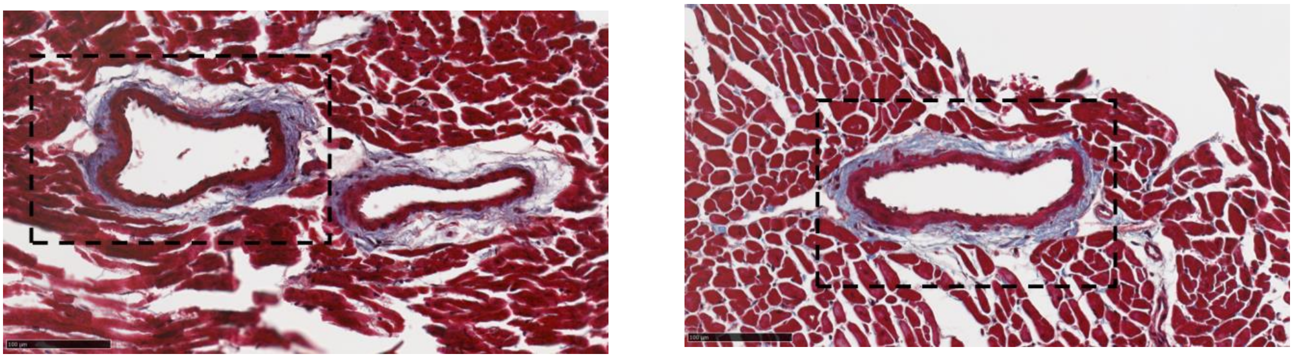

Supplemental Figure S1: Original, full uncropped images of H&E-stained hearts of (**a**) control and (**b**) exercise animals. Black dashed squares denote cropped area used for Figure 2d. Scale bars: 100 µm.

Supplemental Figure S2

(**a**) (**b**)

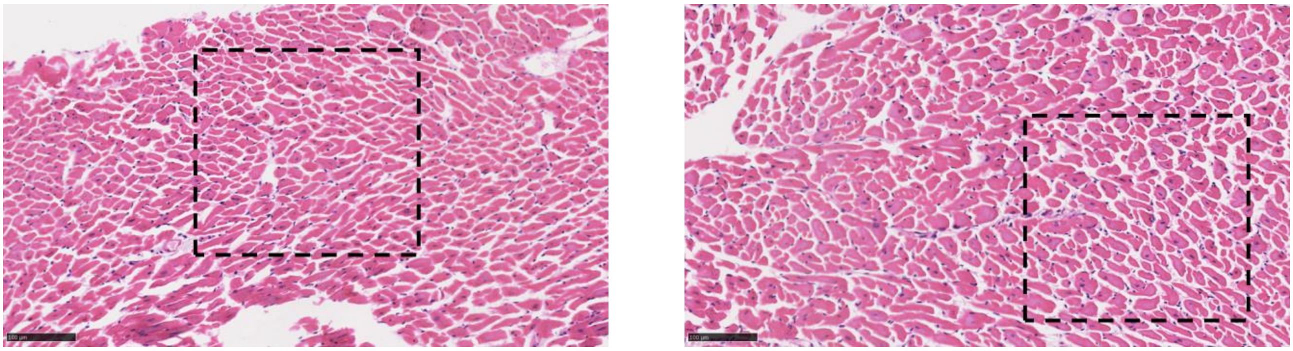

Supplemental Figure S2: Original, full uncropped images of Masson's trichrome-stained hearts depicting collagen (blue) deposition surrounding the vessels of (**a**) control and (**b**) exercise animals**.** Black dashed squares denote cropped area used for Figure 3d. Scale bars: 100 µm.

Supplemental Figure S3

(**a**) (**b**)

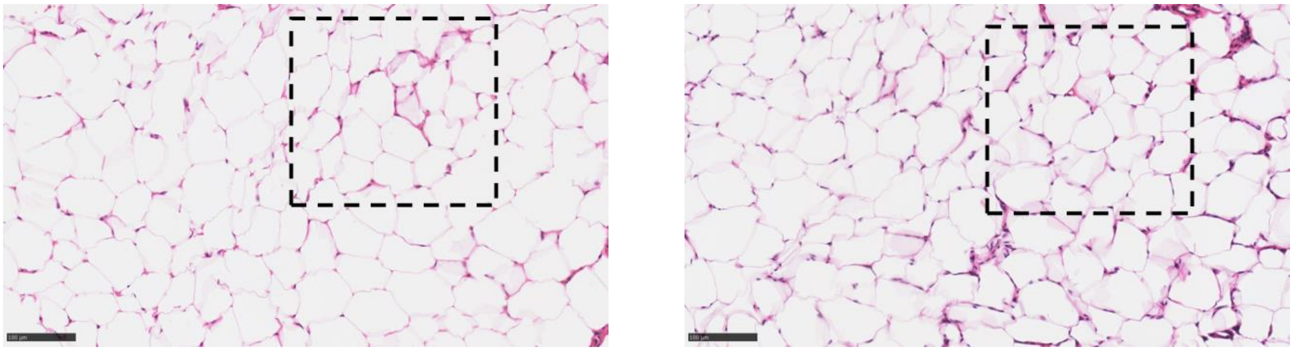

Supplemental Figure S3: Original, full uncropped images of H&E-stained visceral adipose tissues of (**a**) control and (**b**) exercise animals**.** Black dashed squares denote cropped area used for Figure 7a. Scale bars: 100 µm.

Supplemental Figure S4

(**a**) (**b**)

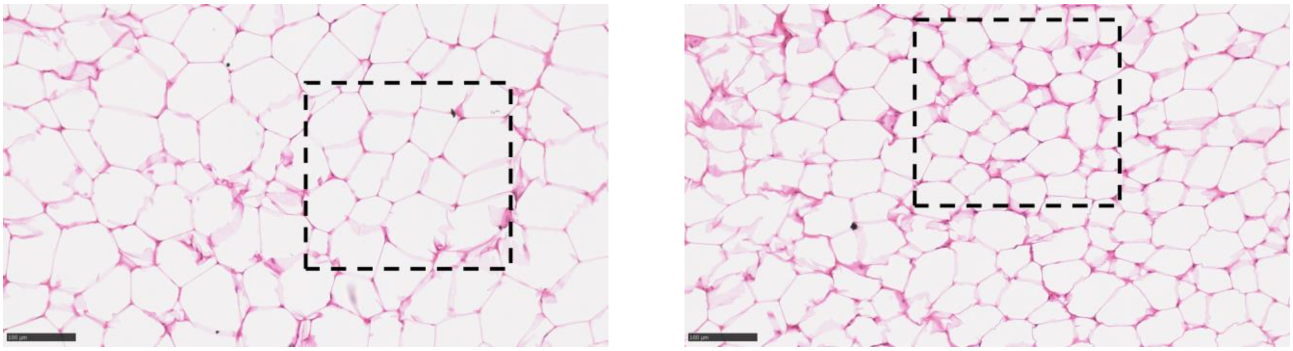

Supplemental Figure S4: Original, full uncropped images of H&E-stained subcutaneous adipose tissues of (**a**) control and (**b**) exercise animals**.** Black dashed squares denote cropped area used for Figure 7b. Scale bars: 100 µm.

Supplemental Figure S5

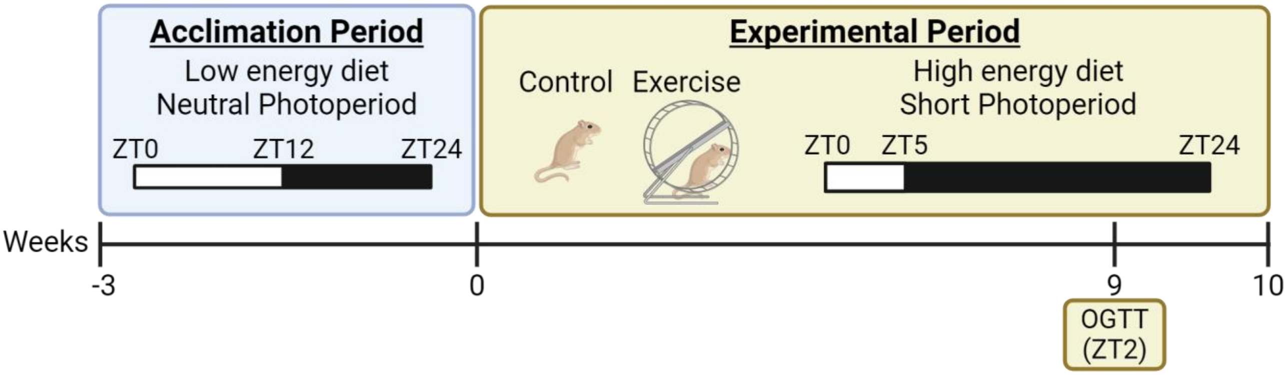

Supplemental Figure S5: HsdHu diabetes-prone male sand rats (*Psammomys obesus*, 6–7 months old) were initially maintained on a low energy diet and neutral photoperiod (12 h light:12 h dark – ZT0: lights on, ZT12: lights off) to prevent the development of diabetes. After 3 weeks of acclimation, the animals were assigned to experimental groups based on the weights and blood glucose levels to avoid baseline bias. Animals were exposed to short photoperiod (5 h light:19 h dark – ZT0: lights on, ZT5: lights off) and a high-energy diet. The Exercise group had voluntary access to a running wheel in their individual cages for 10 weeks. At week 9, animals were subjected to an oral glucose tolerance test at ZT2. At week 10, animals were euthanized around ZT7 (dark phase). Created with BioRender.com.
